# Thinking Green: Sustainable Polymers from Renewable Resources

**DOI:** 10.3390/polym10090952

**Published:** 2018-08-27

**Authors:** George Z. Papageorgiou

**Affiliations:** Department of Chemistry, University of Ioannina, P.O. Box 1186, 45110 Ioannina, Greece; gzpap@uoi.gr

The use of polymeric materials from renewable resources has a long history, with naturally occurring polymers being among the first materials used by men. In the 19th century, natural materials, such as casein, natural rubber, and cellulose, were modified to obtain useful polymeric materials. Over the past few decades, the production and application of synthetic polymers have seen an almost exponential increase. However, concerns regarding depletion of fossil resources, disposal and related issues, as well as government policies, have led to a continuously growing interest in the development of sustainable, safe, and environmentally friendly plastics from renewable resources [1,2].

Sustainable polymers from renewable resources can be obtained through chemical modification of natural polymers, such as starch, cellulose, or chitin [3,4].

Biobased polymers can also be synthesized through a two-step process from biomass (lignin, cellulose, starch, plant oils) [5,6,7].

Traditional (drop-in) monomers such as ethylene, 1,2-ethanediol, terephthalic acid, or novel monomers like lactide, 2,5-furandicarboxylic acid, 1,4-cyclohexane dicarboxylic acid, furfuryl alcohol, or isosorbide can be obtained through chemical or biochemical conversion [8,9]. All the above can then be polymerized to produce biobased plastics. Therefore, biopolyethylene (bio-PE), bio-poly(ethylene terephthalate) (bio-PET), new polymers such as poly(lactic acid) and poly(ethylene 2,5-furandicarboxylate) (PEF), and even thermosetting polymers can be synthesized from renewable monomers and are expected to play a key role in biobased economy in the near future [10,11].

Finally, polymer synthesis can be achieved in plants through photosynthesis using carbon dioxide (CO_2_) or by microorganisms, e.g., synthesis of poly(hydroxy-alkanoate)s [2]. CO_2_ is also used to synthesize nonisocyanate polyurethanes [12,13].

In general, there are four main strategies to arrive at polymers with tailored properties: (a) selection of the most appropriate monomers for homopolymer production; (b) copolymerization; (c) blending; and (d) use of fillers, fibers, and additives to obtain composites. Apart from the commercialization of the abovementioned biobased and recyclable, but nondegradable, homopolymers (PEF, bio-PE, bio-PET), the use of biodegradable polyesters, such as poly(lactic acid) (PLA), poly(β-hydroxybutyrate) (PHB), poly(butylene adipate) (PBA), poly(butylene succinate) (PBS), or poly(ε-caprolactone) is expected to expand in near future due to their favoring life cycle [14,15].

Biocomposites can be prepared by exploiting the characteristics of the above polymers as well as those of lignocellulose fibers or other biobased fillers. Other biomass-derived materials can also be elaborated for the modification of renewable or fossil-based polymers, e.g., internal plasticizers of polyvinylchloride (PVC).

Uses of polymeric materials from biomass include packaging applications, antimicrobial films, fibers, foams, or coatings production as well as applications in medicine and pharmaceutics [16]. Drug delivery systems, such as drug-loaded micro- or nanoparticles, are a topic of particular interest, considering applications of biodegradable and biocompatible polymers from biomass.

## Contents of This Issue

In this issue, the recent developments in biobased polymers toward general and engineering applications are reviewed [17]. The development of antimicrobial films using plant secondary metabolite-derived polymers is also discussed [18]. Trends in PLA and poly(hydroxy-alkanoate)s (PHA) nanocomposites are presented [19], and important features such as crystallization and stereocomplexation of PLA in multiblock copolymers, as well as biodegradation and mechanical properties of PLA in biocomposites, are studied [20,21,22,23].

Details of PEF synthesis applying solid state polymerization are given by Bikiaris and co-workers [24], and the results of structural investigation of the polymorphic forms of this most promising biobased polyester are reported by Maini et al. [25].

Studies on synthesis of poly(butylene 2,5-furandicarboxylate) (PBF)-related copolyesters containing isophthalate units via ring-opening polymerization and also synthesis and characterization of nanocomposites containing bacterial cellulose based on PBF and related copolymers with butylene diglycolate are included in this issue [26,27].

Sanchez-Lopez et al. have prepared renewable polyesters from isosorbide, 2,5-furandicarboxylic acid (FDCA) succinic acid, 1,3-propanediol, and 1,5-pentanediol for coating applications, while copolyesters based on cyclohexanedicarboxylic acid are synthesized and the gas barrier properties of them are evaluated [28,29].

Polymerization of furfuryl alcohol (FA), which is also a biobased monomer derived from lignocellulosic biomass, is also investigated [30].

Starch-based materials play their own role in biobased materials. Thermoplastic potato starch/halloysite nano-biocomposites are prepared and characterized in a paper in this issue [31].

Polyethylene biocomposites for 3D printing, as well as biocomposites of lignocellulosic biomass and recycled PET, are prepared and characterized [32,33]. Biodegradable poly(ε-caprolactone) blends with ionic liquid are studied in regard to their crystallization characteristics [34]. Furthermore, the use of a green binder based on enzymatically polymerized eucalypt kraft lignin for fiberboard applications is tested [35].

Foamed polymeric composite materials based on polyurethane or PHB with lignin or cellulose are prepared and studied. Lipase-catalyzed synthesis, properties, and application of biobased dimer acid cyclocarbonate with potential applications in nonisocyanate polyurethanes are also studied [36,37,38,39].

Referring to improvements in thermoplastics with internal or external plasticization using renewable materials, poly(vinyl chloride) is modified and plasticized by grafting cardanol groups. Polystyrene is also modified with eugenol for liquid crystal orientation [40,41].

Membranes made of porous regenerated cellulose—suitable bioadsorbents for wastewater treatment—are prepared in two modification stages involving oxidation on both sides and then functionalization with polyethylenimine [42].

Pyrolysis is a technique that can be applied to arrive at monomers and starting materials from renewable resources. In this context, Jiang et al. evaluate the effect of glycerol pretreatment on levoglucosan production by fast pyrolysis [43].

The applications of biopolymers in medicine and pharmaceutics are also included in the scope of this issue. The role of hyaluronic acid in promoting the osteogenesis of the human bone morphogenetic protein-2 in an absorbable collagen sponge is investigated by Huang et al. [44].

Moreover, some cases of applications of polymers from renewable resources in drug delivery systems are examined. An example of such a polymer with interest in drug delivery is chitosan. The potential for tailoring drug release rates by changes in the particle engineering of chitosan-based powders is examined, while thiolated chitosan masked microspheres with mesocellular silica foam are proposed for intranasal delivery of paliperidone [45]. Starch-chitosan polyplexes are also tested as carrier for anti-infectives and gene delivery by Yasar et al. [46,47].

Fucoidan is a polysaccharide composed of chemical units that can be specifically recognized by alveolar macrophages. Inhalable fucoidan microparticles combining two antitubercular drugs—isoniazid and rifabutin—are prepared and evaluated [48]. Furthermore, poly(lactic acid) and poly(lactic acid-co-glycolic acid) (PLGA) nanoparticles are extensively used in drug delivery. In this issue, the dual drug delivery of sorafenib and doxorubicin from PLGA and poly(ethylene glycol)-poly(lactic acid-co-glycolic acid) (PEG-PLGA) polymeric nanoparticles is investigated by Babos et al. [49].

Finally, novel isocyanate-modified carrageenans are prepared and characterized as sorbent materials for preconcentration and removal of diclofenac (DCF) and carbamazepine (CBZ) in different aqueous matrices (surface waters and wastewaters) [50].
